# Editorial: Innovative approaches in glioma therapy: exploring new therapeutic frontiers

**DOI:** 10.3389/fnmol.2025.1697606

**Published:** 2025-09-22

**Authors:** Pilar Marcos, Arturo Mangas, Rafael Coveñas

**Affiliations:** ^1^Human Neuroanatomy Laboratory, Instituto de Biomedicina de Castilla-La Mancha, University of Castilla-La Mancha, Albacete, Spain; ^2^Laboratory of Neuroanatomy of the Peptidergic Systems, Institute of Neurosciences of Castilla and León (INCYL), University of Salamanca, Salamanca, Spain; ^3^Group GIR USAL: BMD (Bases Moleculares del Desarrollo), University of Salamanca, Salamanca, Spain

**Keywords:** glioma, ion channels, tumor microenvironment, gut microbiota, clusterin, lysine crotonylation-related long non-coding RNAs, pleomorphic xanthoastrocytoma, signaling pathways

The treatment of glioma, one of the malignant tumor types with the highest mortality rate, unfortunately has very limited therapeutic choices. Therefore, the main aim of this Research Topic is to gather knowledge about glioma to help the development of new lines of research to fight the disease with greater efficacy and lower toxicity—work that is both necessary and urgent. To this end, the participating researchers show the latest advances in the knowledge and treatment of this heterogenous disease and a plethora of data to improve glioma diagnosis and treatment is presented. Accordingly, the researchers have focused their studies on ion channels and the tumor microenvironment (He et al.), the gut microbiota as a potential therapeutic strategy (Qi et al.), the clusterin protein (Xu Q. et al.), lysine crotonylation-related long non-coding RNAs (Song et al.), pleomorphic xanthoastrocytoma (Tian et al.), signaling pathways and metformin (Ma et al.), apoptosis mechanisms by inhibition of ADORA1 (Li et al.), the brain molecule Fyn (Xu C. et al.), combination photodynamic therapy and tumor-treating fields therapy (Fukami et al.), and intraoperative radiotherapy (Wen et al.).

Two groups have highlighted the influence of the tumor microenvironment on glioblastoma and have studied this parameter from different approaches to explore the possibility of making it a therapeutic target. Ion channels play an important role in tumor cells, regulating migration and apoptosis. The group, headed by Dr. Yingzi Liu (The 4^th^ Hospital of Hebei Medical University, Hebei, China), performed a bibliometric analysis of studies from 2005 to 2024 focused on ion channels in glioblastoma (He et al.). They concluded that calcium and chloride channels were the most studied ion channels and that much research has been focusing on the interactions between ion channels and the tumor microenvironment; thus, to study drugs targeting ion channels to alter this microenvironment is a promising research line. The authors also stated that the efficacy of ion channel-targeting treatments can be augmented by integrating single-cell spatial transcriptomics and nanoparticle technologies. On a different note, the group led by Dr. Zhaoqun Feng, from the Neurosurgery Department of the Encephalopathy Hospital, Affiliated Hospital of Shaanxi University of Chinese Medicine (Shaanxi, China), has summarized the role of the gut microbiota in the tumor microenvironment (Qi et al.). The establishment of the gut-brain axis makes it possible that the gut microbiota can influence the microenvironment on glioma survival and progression. Authors describe that this microenvironment regulates the tumor's nutrient supply, growth, immune escape and invasion, and metastasis. Gut microbiota play a dual-regulatory role in tumorigenesis, development, and clinical outcomes by acting as a regulator of the gut-brain axis as well as by driving the malignant progression of glioma. However, the exact mechanisms by which gut microbiota dysbiosis leads to changes in the tumor microenvironment and glioma have not been fully elucidated: the specific molecular mechanisms, signal pathways, or biomarkers involved in this regulation are still unknown. The development of animal models and/or specific biomarkers may be key aspects to consider in the diagnosis and treatment of glioma.

Other papers contributed to this Research Topic are more focused on molecular studies. The group headed by Dr. Xiji Shu and Yiyuan Xia (Jianghan University, Wuhan, China) has demonstrated that the protein clusterin favored glioma progression through BCL2L1-dependent regulation of apoptotic resistance (Xu Q. et al.). They have demonstrated that clusterin is upregulated in glioma and has been associated with poor clinical outcomes and tumor malignancy increase; that clusterin and BCL2L1 favored glioma cell proliferation and migration; that clusterin controlled the expression of BCL2L1, blocking apoptosis; and that clusterin silencing decreased glioma cell proliferation and migration and inhibited glioma growth *in vivo*. The data suggest that the axis clusterin-BCL2L1 is a promising therapeutic target for the treatment of glioma. Dr. Ying Wang and their coworkers (Second Affiliated Hospital of Anhui University of Chinese Medicine, Hefei, China) have reported the characterization of lysine crotonylation-related long non-coding RNAs (LCRlncRNAs) for prognostic assessment and immune response in glioma (Song et al.). The authors identified six LCRlncRNAs showing important prognostic values, stated a risk score model, suggested LCRlncRNAs as glioma biomarkers and therapeutic targets, and offered important data regarding the epigenetic-immune axis underlying treatment resistance. The study integrates multi-omics analysis, clinical samples, and functional experiments and the authors concluded that LCRlncRNAs are associated with immune microenvironment remodeling, glioma prognosis, and therapeutic responses. Moreover, the LCRlncRNA-based risk model reported in this study offers an instrument for prognostic assessment and personalized therapy strategy against glioma. An interesting case report has been presented by Dr. Xiaoying Xue and his group (The Second Hospital of Hebei Medical University, Hebei, China; Tian et al.). They describe a rare and complex malignant transformation and systemic metastasis of pleomorphic xanthoastrocytoma, a rare benign WHO grade II astrocytoma. Nine years after surgery, the tumor recurred as high-grade glioblastoma and CDKN2A homozygous deletion and BRAF V600E mutation were detected. Despite surgery, targeted therapy, and radiotherapy, the tumor spread to the bones and spinal cord, and unfortunately the patient died. The authors concluded that the molecular mechanisms involved in the pleomorphic xanthoastrocytoma malignant transformation need an optimized targeted therapy approach founded on long-term vigilance for distant metastasis and molecular profiling. The study by the group led by Dr. Xingyuan Ma (The First School of Clinical Medicine, Lanzhou University, Lanzhou, China) explored the possibilities of the use of metformin, a drug commonly used against type 2 diabetes, as it has certain inhibitory effects on glioma (Ma et al.). These effects are due to the action of metformin on certain mechanisms such as AMPK/mTOR, ferroptosis, autophagy, and apoptosis. Metformin inhibits certain key signaling pathways such as STAT3, mTOR, and AKT, thus contributing to altering the tumor microenvironment which, as has been suggested, may be an interesting therapeutic target. Although the pathways on which metformin acts are gradually being discovered, many other signaling pathways regulated or influenced by metformin are still unknown. This treatment drug, sometimes in combination with other strategies such as radiation, seems promising especially in treatment-resistant gliomas, although clinical studies on this subject are still limited. On the other hand, the group led by Dr. Hong-Jiang Li (Department of Neurosurgery, The First Affiliated Hospital of Zhengzhou University, Zhengzhou, China) carried out a very comprehensive study, with cell cultures to animal models and patient samples, of an immune checkpoint blockade therapy that proved to be effective against different cancer types (Li et al.). It has been described that the adenosine A1 receptor (ADORA1) facilitates the proliferation of tumors in cancer, and the increase of ADORA1 expression in gliomas is associated with poor prognosis. The authors suggest that inhibiting ADORA1 can induce apoptosis on glioma cells by augmenting kininogen-1 and enhancing T cell recruitment. Moreover, this inhibition can increase the sensitivity of glioma cells to anti-programmed death receptor 1 (PD1) therapy. Thus, the authors propose ADORA1 as a prognostic marker for glioma and suggest that this receptor may also be a potential target to enhance the effectiveness of anti-PD-1 therapy. In another study also carried out at the molecular level, the research group of Dr. Chongxi Xu (Department of Neurosurgery, West China Hospital, Sichuan University, Chengdu, China) suggest that Fyn, a non-receptor tyrosine kinase present in brain, may be a potential therapeutic target in gliomas (Xu C. et al.). Fyn is a member of the Src family of kinases (SFKs) involved in cell morphogenic transformation, motility, proliferation, and death that influences the development and progression of various cancer types since it is expressed aberrantly and extensively in different cell types. Under physiological conditions, Fyn also acts in the formation and activation of T lymphocytes. It has been reported that the inhibition of Fyn, linked to certain signaling molecules specific to tumor cells, can enhance patient outcomes and prolong survival, making this molecule a possible therapeutic target for several types of cancer including gliomas (Xu C. et al.). However, Fyn shows strong similarities with other Src family kinases and is expressed widely throughout the body, thus targeted therapy must be carefully studied to avoid unanticipated and unwanted consequences.

Other authors describe therapies more focused on treatments that are already being applied in the clinic. The study carried out by Dr. Shinjiro Fukami and collaborators (Tokyo Medical University, Tokyo, Japan) reported that the combination of newly diagnosed glioblastoma with photodynamic therapy and tumor-treating fields therapy prolonged the time to recurrence and improved survival outcomes of patients (Fukami et al.). This combination therapy did not show major adverse events, although some problems with the tumor-treating fields therapy continuation were observed and, importantly, it seems that this strategy targets local recurrence. The median progression-free survival was 13.4 months. The promising results must be confirmed in a larger number of patients since the current study was performed with only 14 individuals. The group led by Dr. Zhongcheng Wen (Department of Neurosurgery, General Hospital of Northern Theater Command, Postgraduate Training Base of Dalian Medical University, Shenyang, China) report an interesting single-center retrospective study about the efficacy and safety of low-dose X ray-based intraoperative radiotherapy (IORT) for high-grade gliomas (Wen et al.). IORT is an emerging local therapy in the surgery of intra-axial brain tumors to improve clinical outcomes and accelerate the adjuvant oncologic therapy. In this study, 30 patients diagnosed with high-grade gliomas were recruited to undergo surgical treatment and IORT, with a mean dose of 12 Gy. No severe radiation-related adverse effects were observed, so the authors concluded that these low doses of IORT are safe for this type of patient. Results indicate that patients who received postoperative radiotherapy and chemotherapy after IORT had better clinical outcomes than those who did not and that post-operative radiotherapy was independently correlated with favorable clinical outcomes.

Taken together, the results found in this Research Topic increase knowledge on glioma from different approaches, from the molecular level to the clinical application of various therapies, and include studies in cell culture and/or animal models. This Research Topic opens new research lines and possibilities to improve glioma diagnosis and treatment and explore new therapeutic strategies. We hope that the contributions made to this Research Topic may serve to develop new and more effective therapeutic strategies in the near future to fight glioma effectively.

